# Kinetic Model of Urea-Related Deposit Reactions

**DOI:** 10.3390/molecules28052340

**Published:** 2023-03-03

**Authors:** Neng Zhu, Yu Hong, Feng Qian, Xiaowei Xu

**Affiliations:** School of Automotive and Transportation Engineering, Wuhan University of Science and Technology, Wuhan 430081, China

**Keywords:** urea, deposit, reaction kinetic, thermal analysis technology, selective catalytic reduction

## Abstract

The thermal analysis kinetic method was employed to solve the activation energies of the thermal decomposition reactions of urea and cyanuric acid, with the purpose of understanding the formation of deposits in the diesel engine SCR system. The deposit reaction kinetic model was established by optimizing the reaction paths and reaction kinetic parameters based on the thermal analysis test data of the key components in the deposit. The result shows that the established deposit reaction kinetic model can accurately describe the decomposition process of the key components in the deposit. Compared to the Ebrahimian model, the simulation precision of the established deposit reaction kinetic model is significantly improved above 600 K. The activation energies of the urea and cyanuric acid decomposition reactions are 84 kJ/mol and 152 kJ/mol, respectively, after model parameters identification. The identified activation energies were closest to those of the Friedman one-interval method indicating that the Friedman one-interval method is reasonable to solve the activation energies of deposit reactions.

## 1. Introduction

The need to control harmful gas emissions and improve environmental quality is becoming increasingly strong, with global environmental pollution becoming increasingly prominent and people’s awareness of environmental protection gradually strengthening. Nitrogen oxides (NO*_x_*) are one of the main harmful emissions from diesel engines, which has caused great harm to human health, the ecological environment and the climate. Nowadays, selective catalytic reduction (SCR) systems [1] have been increasingly used in diesel as the mainstream device to deal with NO*_x_* emissions [2,3].

Urea-SCR technology is to spray urea aqueous solution into the exhaust pipe of a diesel engine at a suitable location; it produces the reducing agent NH_3_ after evaporation, pyrolysis and hydrolysis, and NH_3_ converts the harmful NO*_x_* into harmless N_2_ and H_2_O under the action of a catalyst. Numerous studies [4,5,6,7,8] have revealed that the exhaust pipe wall of diesel engines with urea-SCR systems was prone to form deposits consisting of undecomposed urea, biuret, and cyanuric acid (CYA). The deposits easily lead to partial or even total blockage of the exhaust pipe, which increases the exhaust back pressure and seriously affects the performance of diesel engines [9].

The mechanism of the urea pyrolysis reaction is complex. Dong, et al. [10] have studied the pyrolysis process of urea using thermogravimetric combined with Fourier infrared spectroscopy analytical methods. The result indicated that the urea pyrolysis process went through three stages. Each stage occurred at 193 °C, 250 °C and 400 °C, corresponding to residual mass fractions of 46.2%, 39.5% and 9.2%, respectively. The polymerization of HNCO and the condensation reaction with urea and biuret are the main reasons for producing polymeric compounds. Schaber, et al. [11] have studied the thermal decomposition process of urea in detail. The results indicated that the urea pyrolysis process could be divided into four reaction stages, where the first and second stages were the main reaction processes. The mass loss was mainly related to the urea decomposition in the first reaction stage (room temperature to 190 °C). The urea was decomposed into the biuret and began to slowly synthesize complex products, such as cyanuric acid and cyanuric acid monoamide. The urea continued to decompose and the biuret began to decompose at the second reaction stage (190~250 °C). The production rate of cyanuric acid and cyanuric acid monoamide increased, while little cyanuric acid amide and melamine began to be generated. The third reaction stage (250~360 °C) and the fourth reaction stage (>360 °C) were mainly the decomposition and sublimation of residues. Zhao [12] from Tsinghua University studied the influence of the urea pyrolysis process at different temperatures and different heating rates by thermogravimetric tests. Thagard [13] has analyzed the urea pyrolysis process at 150–200 °C using the DBD method. The results indicated there was no difference in the urea pyrolysis by-products whether in wet or dry air, the main gaseous products were NH_3_ and HNCO, and the residual solid product was CYA. In addition, Stradella [14], Carp [15] and Lundström [16] have conducted studies related to urea pyrolysis as well.

**Figure 1:**
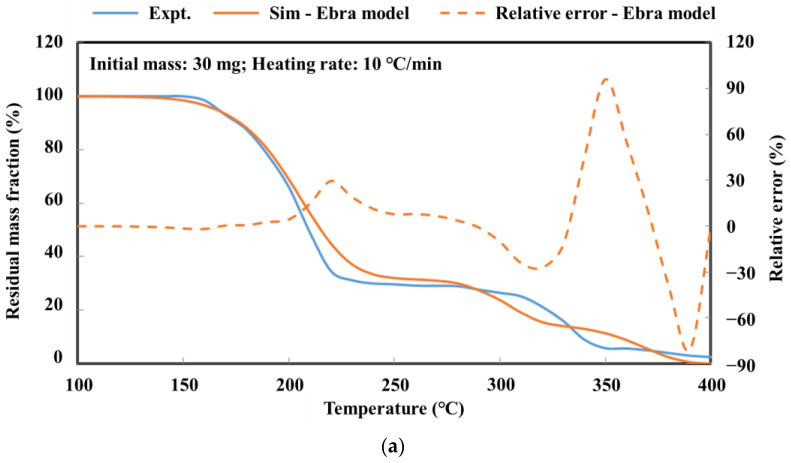

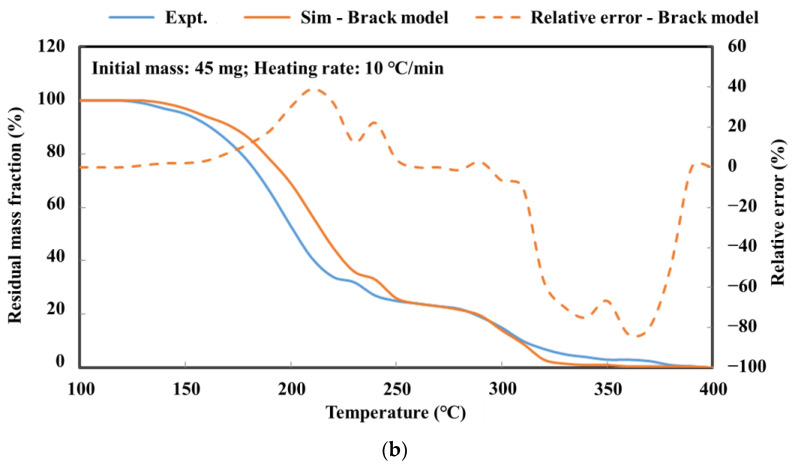
Experimental and simulation results comparison of urea pyrolysis (**a**) Comparison result of Ebrahimian model (**b**) Comparison result of Brack model.

The detailed kinetic model of the deposit reaction is required to quantitatively describe the production of the deposit. Ebrahimian [17] has established the reaction kinetic model of the urea pyrolysis process. It considered four kinds of components of deposit, including urea, biuret, CYA and ammelide, which provided a guide for the quantitative study of deposit formation. Brack, et al. [18] have revised the reaction path based on the Ebrahimian mechanism model. They re-identified the reaction kinetic parameters, according to the results of thermogravimetric tests with different urea initial masses, temperature heating rates and reactor configurations. However, Figure 1 shows that the simulation values of urea pyrolysis, respectively, from the above reaction kinetic models established by Ebrahimian and Brack, are different from the experimental values.

In this study, thermal analysis technology was applied to the investigation of chemical reaction kinetics, and various classical thermal analysis kinetic methods were used to solve the activation energy of the decomposition reactions of urea and CYA. According to the Ebrahimian mechanism model, we re-identified the reaction kinetic parameters and established a deposit reaction kinetic model to describe the decomposition process of the key components in the deposit, attempting to provide a reference for the quantitative study of the deposit formation.

## 2. Results

### 2.1. Solving the Activation Energy of Urea Pyrolysis Reaction

#### 2.1.1. The Flynn–Wall–Ozawa Method

**Figure 2:**
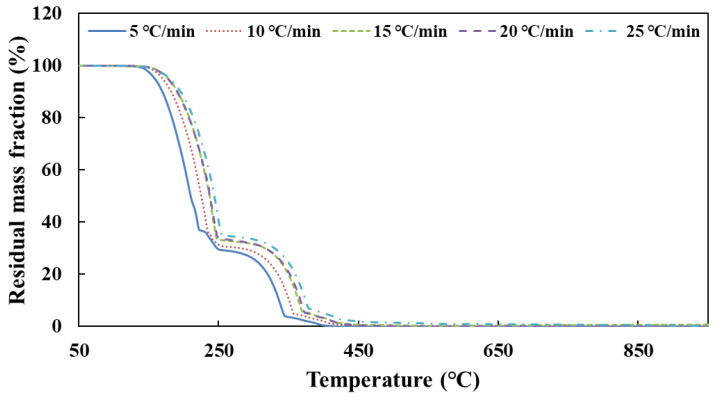
The TG curves of urea pyrolysis at different heating rates.

Figure 2 shows the TG curves of urea pyrolysis at different heating rates.

According to the TG curves of urea pyrolysis, the temperature data corresponding to each conversion rate are obtained at different heating rates. Table 1 shows the result of the activation energy of urea decomposition by substituting these data into Equation (24). *E*_12_ is the result from two sets of data with heating rates of 5 and 10 °C/min, *E*_23_, *E*_34_ and *E*_45_ to follow.

**Figure A1:**
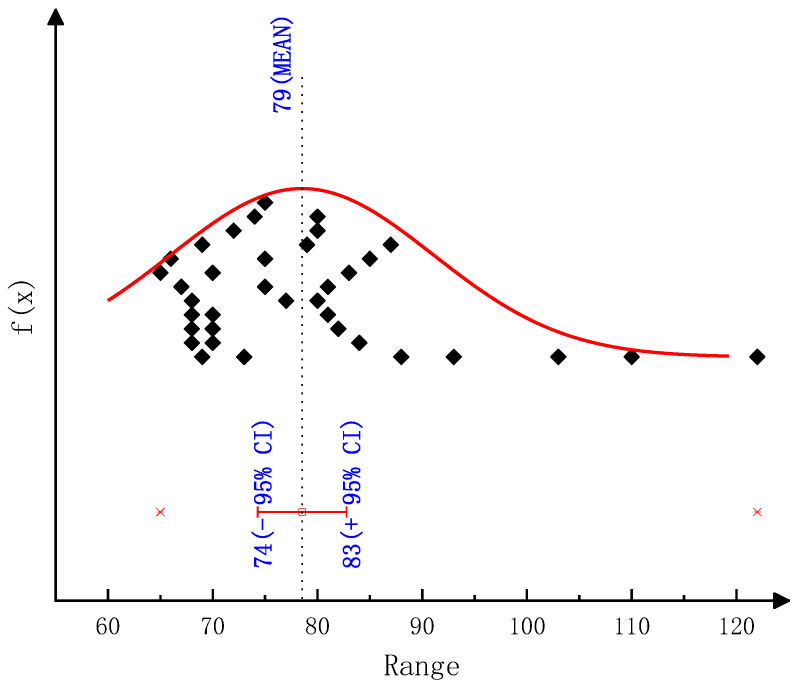
95% confidence interval for the activation energy *E*_O_ of urea pyrolysis reaction.

As the results show in Table 1, *E*_34_ has a large error in the process of calculating the activation energy of urea decomposition. The reason is that the two thermogravimetric curves of heating rates *β*_3_ and *β*_4_ almost coincide. The difference of temperature *T* corresponding to them is very small for the same conversion rate *α*, which brings a large error. Therefore, the set of data was discarded when calculating the total activation energy *E*. Finally, the activation energy of urea decomposition is 79 kJ/mol (95% confidence interval, CI: 74–83, as shown in Figure A1) calculated from the Ozawa method.

#### 2.1.2. The Friedman-Reich-Levi Method

According to the TG curves of urea pyrolysis, d*α*/d*T* corresponding to each conversion rate is obtained at different heating rates. These data are substituted into Equation (28) to obtain the results of the activation energy of urea decomposition from the two-interval method (as shown in Table 2) and the one-interval method (as shown in Table 3).

**Figure A2:**
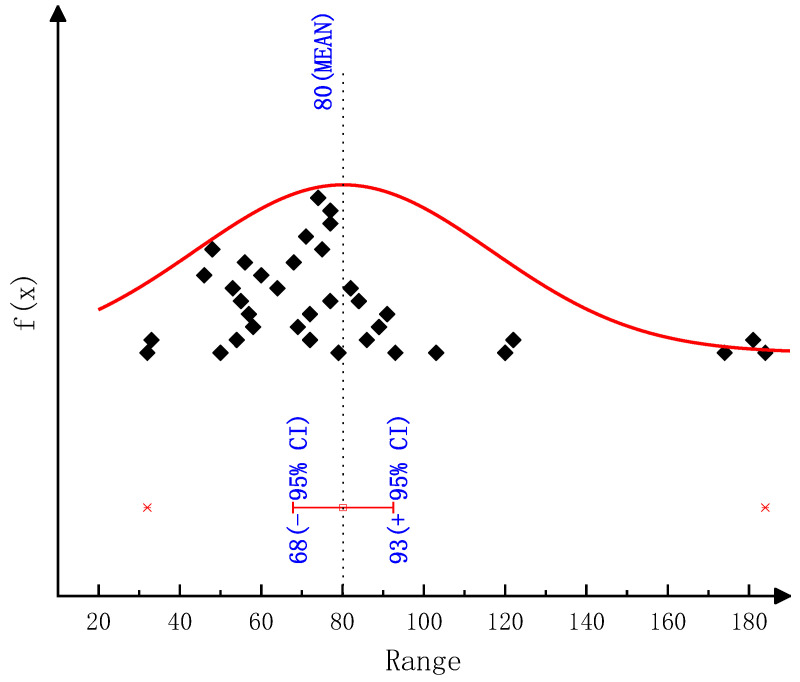
95% confidence interval for the activation energy *E*_F,2_ of urea pyrolysis reaction.

**Figure A3:**
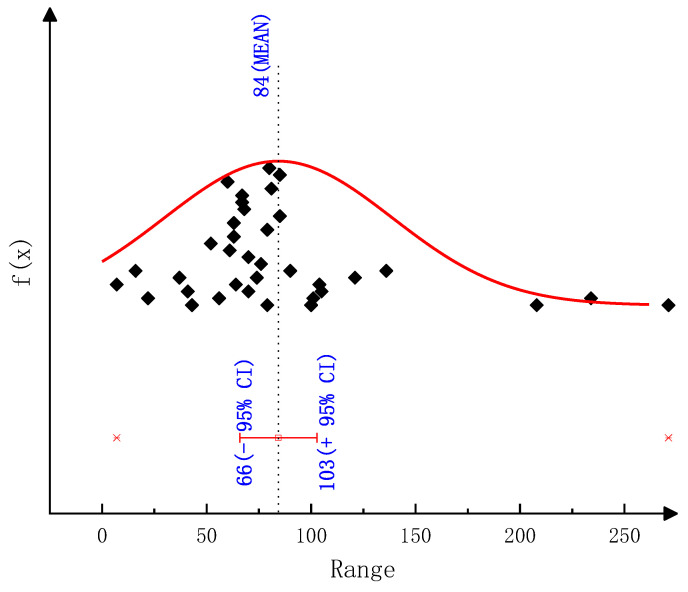
95% confidence interval for the activation energy *E*_F,1_ of urea pyrolysis reaction.

As the results show in Table 2 and Table 3, *E*_34_ has a large error in the process of calculating the activation energy of urea decomposition. The reason is that the two thermogravimetric curves of heating rates *β*_3_ and *β*_4_ almost coincide. The difference between temperature *T* and d*α*/d*T* corresponding to them is very small for the same conversion rate *α*, which brings a large error. Therefore, the set of data was discarded when calculating the total activation energy *E*. Finally, the activation energy of urea decomposition is 80 kJ/mol (95% confidence interval, CI: 68–93, as shown in Figure A2) calculated from the Friedman two-interval method and 84 kJ/mol (95% confidence interval, CI: 66–103, as shown in Figure A3) calculating from the Friedman one-interval method.

#### 2.1.3. The Kissinger–Akahira–Sunose Method

**Figure 3:**
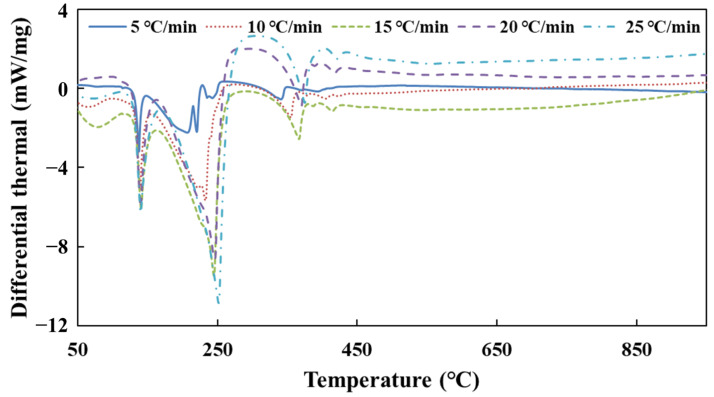
The DSC curves of urea pyrolysis at different heating rates.

Figure 3 shows the DSC curves of urea pyrolysis at different heating rates.

According to the DSC curves of urea pyrolysis, the peak temperature date *T_p_* is obtained at different heating rates. Table 4 shows the result of the activation energy of urea decomposition by substituting these data into Equation (32).

**Figure A4:**
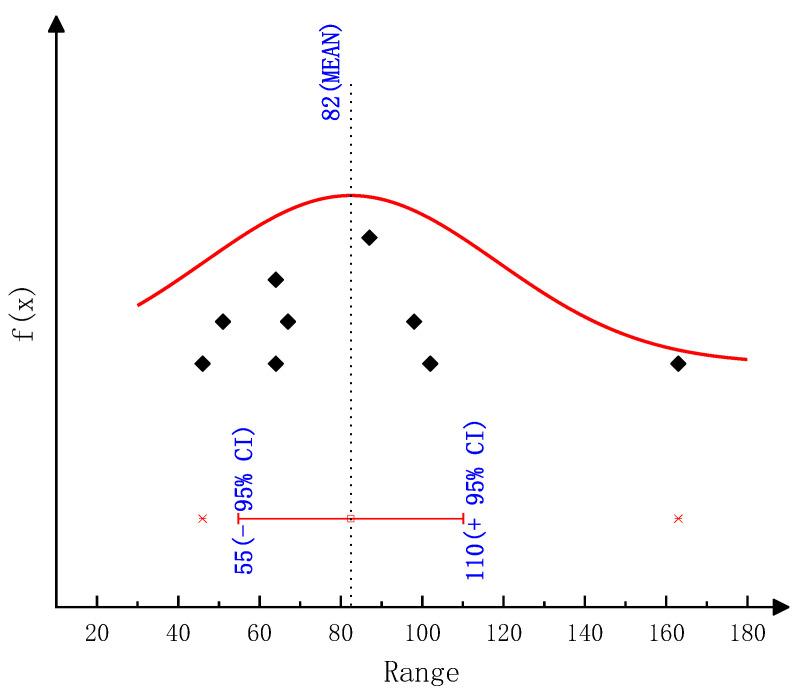
95% confidence interval for the activation energy *E*_K_ of urea pyrolysis reaction.

As the results show in Table 1, *E*_34_ has a large error in the process of calculating the activation energy of urea decomposition. The reason is that the difference between the peak temperatures *T_p_* on the two DSC curves of heating rates *β*_3_ and *β*_4_ is very small, which brings a large error. Therefore, the set of data was discarded when calculating the total activation energy *E*. Finally, the activation energy of urea decomposition is 82 kJ/mol (95% confidence interval, CI: 55–110, as shown in Figure A4) calculated from the Kissinger method.

### 2.2. Solving the Activation Energy of Cyanuric Acid (CYA) Pyrolysis Reaction

#### 2.2.1. The Flynn–Wall–Ozawa Method

**Figure 4:**
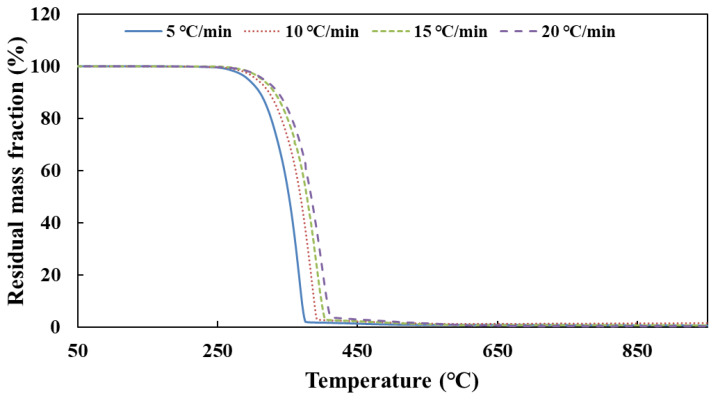
The TG curves of CYA pyrolysis at different heating rates.

Figure 4 shows the TG curves of CYA pyrolysis at different heating rates.

**Figure A5:**
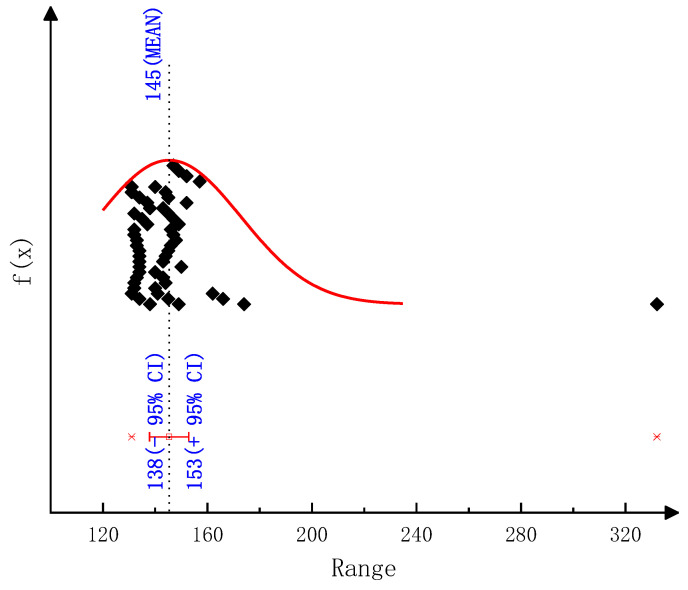
95% confidence interval for the activation energy *E*_O_ of CYA pyrolysis reaction.

According to the TG curves of CYA pyrolysis, the temperature data corresponding to each conversion rate are obtained at different heating rates. Table 5 shows the result of the activation energy of CYA decomposition by substituting these data into Equation (24). Finally, the activation energy of CYA decomposition is 145 kJ/mol (95% confidence interval, CI: 138–153, as shown in Figure A5) calculated from the Ozawa method.

#### 2.2.2. The Friedman–Reich–Levi Method

**Figure A6:**
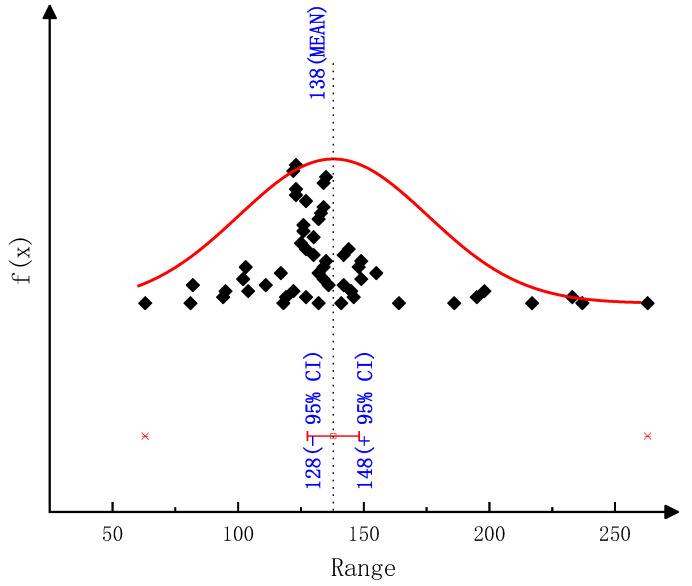
95% confidence interval for the activation energy *E*_F,2_ of CYA pyrolysis reaction.

**Figure A7:**
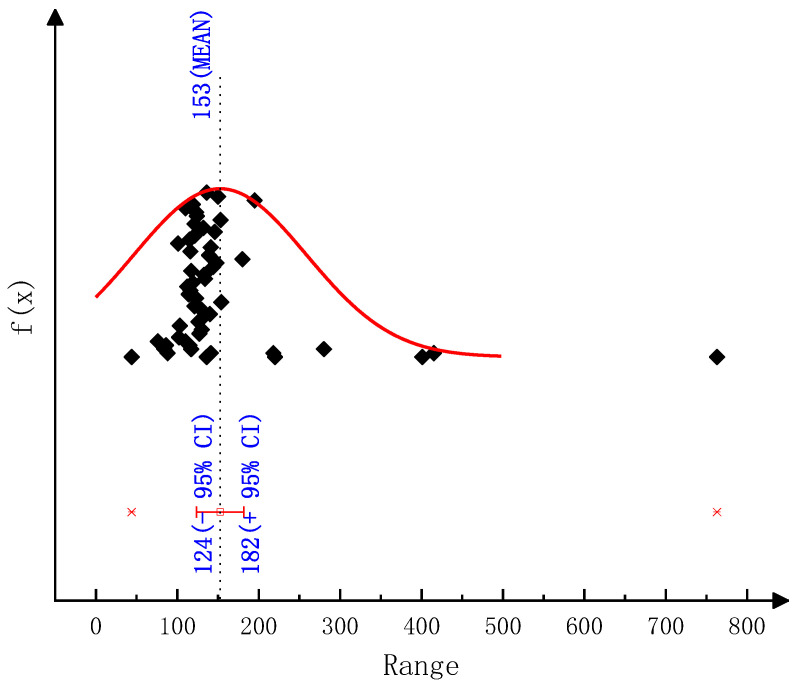
95% confidence interval for the activation energy *E*_F,1_ of CYA pyrolysis reaction.

According to the TG curves of CYA pyrolysis, d*α*/d*T* corresponding to each conversion rate is obtained at different heating rates. These data are substituted into Equation (28) to obtain the results of the activation energy of CYA decomposition from the two-interval method (as shown in Table 6) and the one-interval method (as shown in Table 7). Finally, the activation energy of CYA decomposition is 138 kJ/mol (95% confidence interval, CI: 128–148, as shown in Figure A6) calculated from the Friedman two-interval method and 153 kJ/mol (95% confidence interval, CI: 124–182, as shown in Figure A7) calculated from the Friedman one-interval method.

#### 2.2.3. The Kissinger–Akahira–Sunose Method

**Figure 5:**
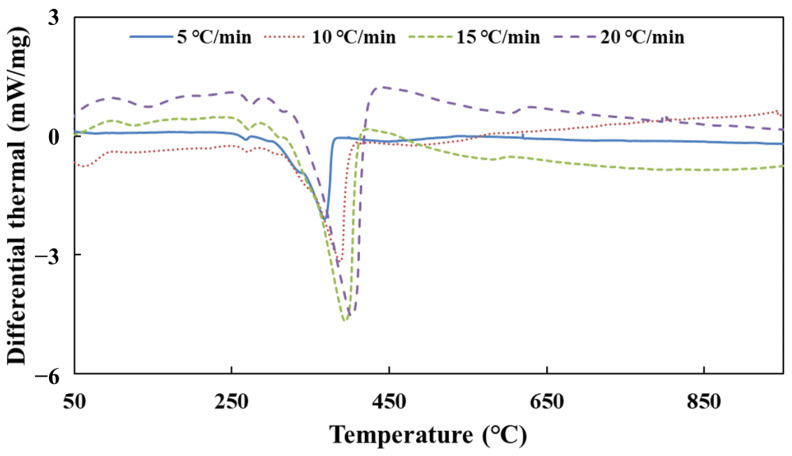
The DSC curves of CYA pyrolysis at different heating rates.

Figure 5 shows the DSC curves of CYA pyrolysis at different heating rates.

**Figure A8:**
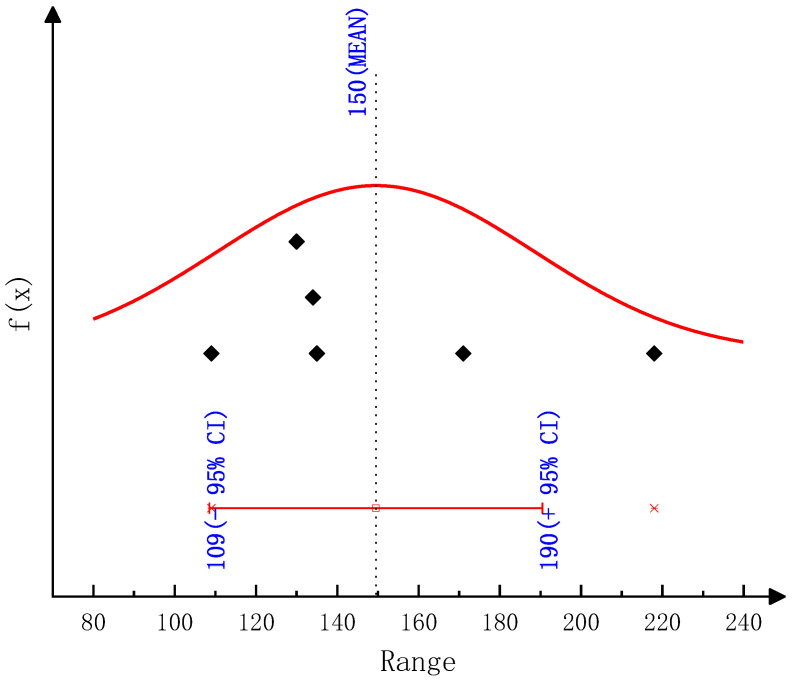
95% confidence interval for the activation energy *E*_K_ of CYA pyrolysis reaction.

According to the DSC curves of CYA pyrolysis, the peak temperature data *T_p_* is obtained at different heating rates. Table 8 shows the result of the activation energy of CYA decomposition by substituting these data into Equation (32). Finally, the activation energy of CYA decomposition is 150 kJ/mol (95% confidence interval, CI: 109–190, as shown in Figure A8) calculated from the Kissinger method.

### 2.3. Kinetic Modeling of Deposit Reaction

#### 2.3.1. Reaction Path

Urea, biuret, CYA and ammelide are the four components of deposits. Equations (1)–(12) show the reaction paths of urea pyrolysis given by Ebrahimian.

R1            Urea → NH_4_^+^ + NCO^−^ (1)

R2               NH_4_^+^ → NH_3_ + H^+^ (2)

R3             NCO^−^ + H^+^ → HNCO (3)

R4           Urea + NCO^−^ + H^+^ → Biuret (4)

R5           Biuret → Urea + NCO^−^ + H^+^ (5)

R6        Biuret + NCO^−^ + H^+^ → CYA + NH_3_ (6)

R7              CYA → 3NCO^−^ + 3H^+^ (7)

R8     CYA + NCO^−^ + H^+^ → Ammelide + CO_2_ (8)

R9    Ammelide → 2NCO^−^ + 2H^+^ + HCN + NH (9)

R10             Urea (aq) → NH_4_^+^ + NCO^−^ (10)

R11      NCO^−^ + H^+^ + H_2_O (aq) → NH_3_ + CO_2_ (11)

R12         Urea (aq) + NCO^−^ + H^+^ → Biuret (12)

However, the above mechanism model does not distinguish the different shapes of urea from the perspective of deposit formation in the diesel SCR system. Therefore, the additional reaction paths as shown in Equations (13) and (14) are proposed for the transformation of different urea forms based on the Ebrahimian mechanism model. Moreover, the mechanism model of deposit reaction is established according to Equations (1)–(14).

R13            Urea (aq) → Urea [Dying](13)

R14      Urea (aq) → Urea [Crystallization](14)

#### 2.3.2. Reaction Rate Equation

The generation rate of the component *k* can be expressed as follows [19] for the reaction R1–R12.

(15) $$r_{k}^{\mathrm{reaction}}=\sum_{i=1}^{Nreactions}\nu_{ki}{A^{\prime }}_{i}\exp(-\frac{E_{a,i}}{RT})\prod_{j=1}^{Nspecies}Cs_{j}^{\nu_{ji}}$$

where: *ν_ki_* is the stoichiometric coefficient of the component *k* in the *i*-step reaction; $A_{i}^{’}$ is the reaction pre-exponential factor; *E_a,i_* is the reaction activation energy; *Cs_j_* is the surface concentration of the component *j*.

$A_{i}^{’}$ can be calculated by the following equation:

(16) $${A^{\prime }}_{i}=\frac{A_{i}}{\Gamma^{ni-1}}$$

where: Γ is the active site density; *ni* is the number of activity levels.

The active surface can be calculated by the following equation, assuming the effective area of the model does not change during the whole calculation process.

(17) $$S=\sum_{k=1}^{Nspecies}\frac{m_{k}^{\mathrm{initial}}\sigma_{k}}{W_{k}\Gamma }$$

where: *σ_k_* is the active site occupied by component *k*; *W_k_* is the molecular mass of the component *k*.

*Cs_j_* can be calculated by the following equation:

(18) $$Cs_{j}=\frac{m_{j}}{S\cdot W_{j}}$$

The generation rate of component *k* can be expressed as follows for the reaction R13.

(19) $$r_{k}^{\mathrm{dying}}=K^{\mathrm{dying}}\exp(-\frac{A^{\mathrm{dying}}f_{H_{2}O}}{f_{H_{2}O}^{\max}})Cs_{\mathrm{urea}\_\mathrm{aq}}$$

where: *K*^dying^ and *A*^dying^ are coefficients; *f*_H2O_ is the component concentration of water in the aqueous urea solution; $f_{H_{2}O}^{\max}$ takes the value of 0.876.

The generation rate of component *k* can be expressed as follows for the reaction R14.

(20) $$r_{k}^{\mathrm{cry}}=K^{\mathrm{cry}}\exp[A^{\mathrm{cry}}(T-C^{\mathrm{cry}})]\cdot (w_{\mathrm{urea}}-w_{\mathrm{miller}})$$

where: *K*^cry^ and *A*^cry^ are coefficients; *C*^cry^ takes the value of 233.4 K.

#### 2.3.3. The Model Parameters Identification and Validation

The kinetic parameters of the deposit reaction model were identified, according to the results of thermal analysis experiments for urea, biuret, and CYA. The initial values of the kinetic parameters for the reactions R1–R12 are referred to in the Ebrahimian mechanism model. The value range of activation energy is set to 78–85 kJ/mol for the urea decomposition reaction R1 and 137–153 kJ/mol for the CYA decomposition reaction R7, referring to the results in Section 3.1 and Section 3.2. Table 9 shows the identification results of reaction kinetic parameters. It can be observed that the activation energy of the urea decomposition reaction is 84 kJ/mol and CYA is 152 kJ/mol after identification. Both of the identified activation energies are closest to the results of the Friedman one-interval method.

**Figure 6:**
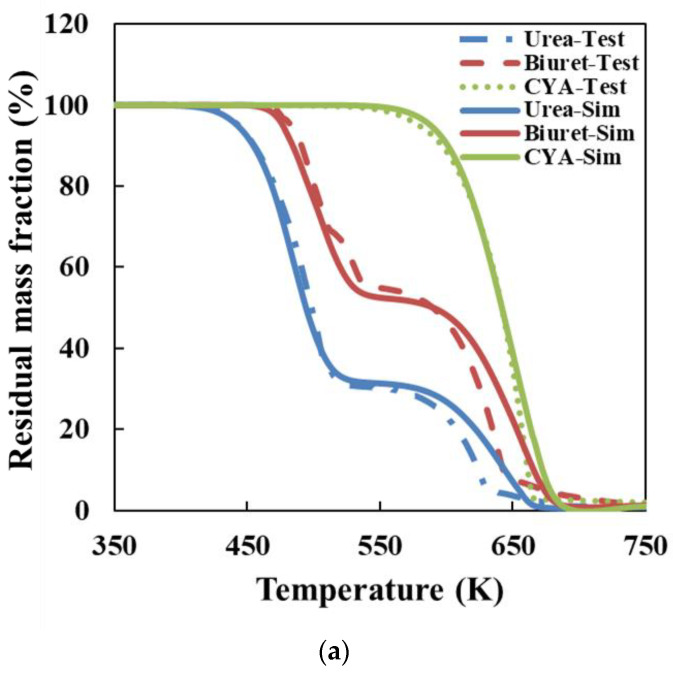

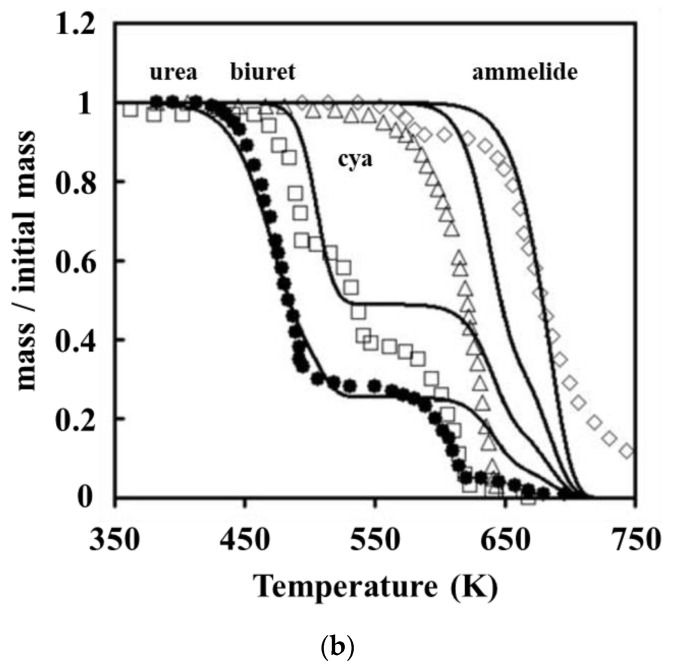
Comparison of the simulation and experiment results of the deposit reaction kinetic model (**a**) Model in this paper (**b**) Ebrahimian model. (Solid line-simulation, symbol-test).

Figure 6a shows the simulation results of the deposit reaction kinetic model in this paper and the thermogravimetric experimental results. The model in this paper can describe the key components of deposit decomposition accurately. Ebrahimian also obtained the comparison of thermogravimetric test and simulation results for each component of the deposits, as shown in Figure 6b. In the Ebrahimian model, the simulation results have an appreciable error above 600 K, which is the initiation temperature of CYA decomposition. We have solved the chemical reaction activation energy of CYA decomposition through the thermogravimetric test. It is employed to constrain the value of parameter identification and enhance the simulation accuracy of the deposit reaction kinetic model over 600 K.

## 3. Materials and Methods

### 3.1. Test Equipment

The integrated thermal analyzer STA449F3 made by German NETZSCH company was employed to simultaneously measure the mass and energy difference curves of the sample with temperature or time, which is to say the TG and DSC curves.

### 3.2. Test Sample

The purity of urea used in the test was not less than 99%, which was provided by Tianjin Guangfu Technology Development Company Limited.

### 3.3. Test Conditions

Each test sample was pulverized into powder form in an agate mortar, and about 10 mg of the sample was placed in an alumina crucible (3 mm in diameter). The purge gas in the heating furnace was Ar with a flow rate of 40 mL/min. The samples were heated from room temperature to 1000 °C at the heating rate of 2, 5, 10, 15, 20, and 25 °C/min.

### 3.4. Kinetic Analysis Method of Thermal Analysis Curves

#### 3.4.1. The Flynn–Wall–Ozawa Method

The Ozawa equation is as follows [20,21]:

(21) $$\ln\beta =\ln\left(\frac{AE}{RG(\alpha )}\right)-5.3308-1.0516\frac{E}{RT}$$

where: *β* is the heating rate (generally constant); *α* is the conversion rate; *A* is the pre-exponential factor; *E* is the activation energy; *R* is the molar gas constant; *T* is the thermodynamic temperature.

The intersection points (*α*, *T*_1_, *β*_1_) and (*α*, *T*_2_, *β*_2_) with the same conversion rate *α* on the two TG curves of different heating rates *β*_1_ and *β*_2_ are substituted into Equation (21) to obtain:

(22) $$\ln\beta_{1}=\ln\left(\frac{AE}{RG(\alpha )}\right)-5.3308-1.0516\frac{E}{RT_{1}}$$

(23) $$\ln\beta_{2}=\ln\left(\frac{AE}{RG(\alpha )}\right)-5.3308-1.0516\frac{E}{RT_{2}}$$

Subtracting Equation (23) from Equation (22) to obtain:

(24) $$\ln\frac{\beta_{1}}{\beta_{2}}=\frac{E}{R}1.0516\frac{T_{1}-T_{2}}{T_{1}T_{2}}$$

The values of *α* are usually 0.95, 0.90, 0.85, …, 0.15, 0.10. An *α* can solve a value of *E*, and the reasonable activation energy *E* can be eventually determined by analyzing all the solved *E* values logically.

#### 3.4.2. The Friedman–Reich–Levi Method

The Friedman equation is as follows [22,23]:

(25) $$\ln(\frac{\beta d\alpha }{dT})=\ln[Af(\alpha )]-\frac{E}{RT}$$

The intersection points (*α*, *T*_1_, (d*α*/d*T*)_1_, *β*_1_) and (*α*, *T*_2_, (d*α*/d*T*)_2_, *β*_2_) with the same conversion rate *α* on the two TG curves of different heating rates *β*_1_ and *β*_2_ are substituted into Equation (25) to obtain:

(26) $$\ln\left[\beta_{1}{\left(\frac{d\alpha }{dT}\right)}_{1}\right]=\ln\left[Af(\alpha )\right]-\frac{E}{RT_{1}}$$

(27) $$\ln\left[\beta_{2}{\left(\frac{d\alpha }{dT}\right)}_{2}\right]=\ln\left[Af(\alpha )\right]-\frac{E}{RT_{2}}$$

Subtracting Equation (27) from Equation (26) to obtain:

(28) $$\ln\left[\frac{\beta_{1}{\left(\frac{d\alpha }{dT}\right)}_{1}}{\beta_{2}{\left(\frac{d\alpha }{dT}\right)}_{2}}\right]=\frac{E}{R}(\frac{T_{1}-T_{2}}{T_{1}T_{2}})$$

The values of *α* are usually 0.95, 0.90, 0.85, …, 0.15, 0.10. An *α* can solve a value of *E*, and the reasonable activation energy *E* can be finally determined by analyzing all the solved *E* values logically.

There are two ways to calculate Δ*α*/Δ*T*, assuming d*α*/d*T* ≈ Δ*α*/Δ*T* and taking the three adjacent points (*α*_1_, *T*_1_), (*α*_2_, *T*_2_) and (*α*_3_, *T*_3_) when processing the experimental data. For point (*α*_2_, *T*_2_), there are:

(i) Two-interval calculation method: d*α*/d*T* ≈ Δ*α*/Δ*T* = (*α*_3_ − *α*_1_)/(*T*_3_ − *T*_1_);
(ii) One-interval calculation method: The points (*α*_1_, *T*_1_) and (*α*_3_, *T*_3_) are averaged to obtain the new point (*α*′, *T*′), then d*α*/d*T* ≈ Δ*α*/Δ*T* = (*α*_2_ − *α*′)/(*T*_2_ − *T*′).

#### 3.4.3. The Kissinger–Akahira–Sunose Method

The Kissinger equation is as follows [24]:

(29) $$\ln(\frac{\beta_{i}}{T_{pi}^{2}})=\ln\frac{AR}{E}-\frac{E}{R}\frac{1}{T_{pi}}(i=1,2,\cdots )$$

The points (*T_p_*_1_, *β*_1_) and (*T_p_*_2_, *β*_2_) at the peak temperature *T_p_* on the two DSC curves of different heating rates *β*_1_ and *β*_2_ are substituted into Equation (29) to obtain:

(30) $$\ln(\frac{\beta_{1}}{T_{p1}^{2}})=\ln\frac{AR}{E}-\frac{E}{R}\frac{1}{T_{p1}}$$

(31) $$\ln(\frac{\beta_{2}}{T_{p2}^{2}})=\ln\frac{AR}{E}-\frac{E}{R}\frac{1}{T_{p2}}$$

Subtracting Equation (31) from Equation (30) to obtain:

(32) $$\ln(\frac{\beta_{1}T_{p2}^{2}}{\beta_{2}T_{p1}^{2}})=\frac{E}{R}(\frac{T_{p1}-T_{p2}}{T_{p1}T_{p2}})$$

According to Equation (32), any two DSC curves can solve a value of *E*. All the solved *E* values are analyzed logically, and the reasonable activation energy *E* can be finally determined.

## 4. Conclusions

According to the thermogravimetric test results, we employed various classical thermal analysis kinetic methods to solve the activation energies of the thermal decomposition reactions of urea and CYA. The activation energy of the urea decomposition reaction: the result is 78.44 kJ/mol by the Ozawa method, 80.12 kJ/mol by the Friedman two-interval method, 84.34 kJ/mol by the Friedman one-interval method and 82.64 kJ/mol by the Kissinger method. The activation energy of CYA decomposition reaction: the result is 145.38 kJ/mol by the Ozawa method, 137.83 kJ/mol by the Friedman two-interval method, 152.57 kJ/mol by the Friedman one-interval method and 149.34 kJ/mol by the Kissinger method. After identifying the reaction kinetic parameters in the model of this paper, the activation energies of the decomposition reactions of urea and CYA are 84 kJ/mol and 152 kJ/mol, respectively. The established deposit reaction kinetic model can accurately describe the decomposition process of each key component of deposits. What is more, the simulation accuracy is significantly improved above 600 K, which can provide a reference for the quantitative study of deposit formation.

## Figures and Tables

**Table 1:** The activation energy *E*_O_ of urea decomposition (Unit: kJ/mol).

1 − *α*	*E*_12_	*E*_23_	*E*_34_	*E*_45_	$\bar{E}$	*E*_O_
0.95	93	80	−597	122	98	79
0.90	88	73	−632	75	79	
0.85	84	70	−625	67	74	
0.80	82	69	−764	65	72	
0.75	81	68	−1528	66	72	
0.70	80	68	−7325	70	73	
0.65	81	70	1484	69	73	
0.60	83	70	657	75	76	
0.55	85	68	511	79	77	
0.50	87	68	5239	72	76	
0.45	103	77	514	74	85	
0.40	110	80	420	75	88	

**Table 2:** The activation energy *E*_F,2_ of urea decomposition from two-interval method (Unit: kJ/mol).

1 − *α*	*E*_12_	*E*_23_	*E*_34_	*E*_45_	$\bar{E}$	*E*_F,2_
0.95	93	50	−539	53	65	80
0.90	79	64	−696	32	58	
0.85	72	54	−664	46	57	
0.80	69	60	−736	56	62	
0.75	72	58	−1416	77	69	
0.70	77	68	−6316	74	73	
0.65	86	75	912	33	65	
0.60	89	57	482	174	107	
0.55	91	55	388	181	109	
0.50	103	71	7189	48	74	
0.45	184	122	260	84	130	
0.40	120	77	494	82	93	

**Table 3:** The activation energy *E*_F,1_ of urea decomposition from one-interval method (Unit: kJ/mol).

1 − *α*	*E*_12_	*E*_23_	*E*_34_	*E*_45_	$\bar{E}$	*E*_F,1_
0.95	79	70	−432	67	72	84
0.90	56	61	−696	22	46	
0.85	70	52	−858	41	54	
0.80	64	63	−1241	7	45	
0.75	74	79	−614	81	78	
0.70	100	63	−7976	60	74	
0.65	90	85	559	85	87	
0.60	76	68	450	37	60	
0.55	101	43	401	16	53	
0.50	105	67	6323	121	98	
0.45	208	271	−1289	136	205	
0.40	104	234	−574	80	139	

**Table 4:** The activation energy *E*_K_ of urea decomposition (Unit: kJ/mol).

Parameter	Value
*E*_12_	46
*E*_13_	51
*E*_14_	64
*E*_15_	67
*E*_23_	64
*E*_24_	102
*E*_25_	98
*E*_34_	431
*E*_35_	163
*E*_45_	87
*E*_K_	82

**Table 5:** The activation energy *E*_O_ of CYA decomposition (Unit: kJ/mol).

1 − *α*	*E*_12_	*E*_23_	*E*_34_	$\bar{E}$	*E*_O_
0.95	149	140	332	207	145
0.90	145	144	174	154	
0.85	141	143	152	145	
0.80	138	140	145	141	
0.75	134	137	144	138	
0.70	131	150	132	138	
0.65	132	143	140	138	
0.60	132	144	166	147	
0.55	133	145	162	147	
0.50	134	146	157	146	
0.45	134	148	152	145	
0.40	134	147	149	143	
0.35	134	146	147	142	
0.30	134	149	138	140	
0.25	133	147	137	139	
0.20	133	145	134	137	
0.15	132	143	131	135	
0.10	132	135	131	133	

**Table 6:** The activation energy *E*_F,2_ of CYA decomposition from two-interval method (Unit: kJ/mol).

1 − *α*	*E*_12_	*E*_23_	*E*_34_	$\bar{E}$	*E*_F,2_
0.95	132	146	195	158	138
0.90	141	145	123	136	
0.85	127	133	117	126	
0.80	118	134	134	129	
0.75	119	127	144	130	
0.70	122	186	263	190	
0.65	136	142	135	138	
0.60	134	149	237	173	
0.55	132	155	122	136	
0.50	134	148	233	172	
0.45	135	104	164	134	
0.40	130	149	217	165	
0.35	127	81	198	135	
0.30	125	142	94	120	
0.25	130	111	123	121	
0.20	126	123	95	115	
0.15	126	102	82	103	
0.10	132	63	103	99	

**Table 7:** The activation energy *E*_F,1_ of CYA decomposition from one-interval method (Unit: kJ/mol).

1 − *α*	*E*_12_	*E*_23_	*E*_34_	$\bar{E}$	*E*_F,1_
0.95	136	112	401	216	153
0.90	141	119	146	135	
0.85	117	134	132	128	
0.80	115	133	121	123	
0.75	110	117	153	127	
0.70	102	220	124	149	
0.65	127	144	123	131	
0.60	130	148	763	347	
0.55	103	180	110	131	
0.50	126	139	119	128	
0.45	131	116	195	147	
0.40	140	141	84	122	
0.35	130	44	280	151	
0.30	121	218	86	142	
0.25	154	88	150	131	
0.20	123	101	136	120	
0.15	114	115	76	102	
0.10	116	121	415	217	

**Table 8:** The activation energy *E*_K_ of CYA decomposition (Unit: kJ/mol).

Parameter	Value
*E*_12_	109
*E*_13_	135
*E*_14_	134
*E*_23_	218
*E*_24_	171
*E*_34_	130
*E*_K_	150

**Table 9:** The identification results of reaction kinetic parameters.

Reaction	Initial		After Identification	
	*E* (kJ/mol)	*A* (s^−1^)	*E* (kJ/mol)	*A* (s^−1^)
R1	84	8.50 × 10^6^	84	8.71 × 10^6^
R2	40	1.50 × 10^2^	40	1.91 × 10^2^
R3	10	6.57 × 10^2^	10	6.30 × 10^2^
R4	115	7.87 × 10^14^	100	8.01 × 10^14^
R5	250	1.50 × 10^24^	243	2.28 × 10^24^
R6	150	2.81 × 10^18^	144	2.83 × 10^18^
R7	260	1.50 × 10^19^	152	2.50 × 10^10^
R8	35	3.48 × 10^5^	36	3.35 × 10^5^
R9	220	6.00 × 10^14^	212	5.67 × 10^14^
R10	84	1.20 × 10^8^	84	1.20 × 10^8^
R11	59	5.62 × 10^9^	59	5.62 × 10^9^
R12	115	3.93 × 10^14^	115	3.93 × 10^14^
R13	*Dying_K*	*Dying_A*	*Dying_K*	*Dying_A*
	1	100	1	100
R14	*Cry_K*	*Cry_A*	*Cry_K*	*Cry_A*
	1	−0.000 5	1	−0.000 5

## Data Availability

No new data were created or analyzed in this study. Data sharing is not applicable to this article.

## Sample Availability

Samples of the compounds are available from the authors.
